# ﻿A new species of *Floronia* Simon, 1887 from Baiyan Cave in Guizhou Province, China (Araneae, Linyphiidae)

**DOI:** 10.3897/zookeys.1185.109285

**Published:** 2023-12-01

**Authors:** Guchun Zhou, Weifeng Du, Chengxiang Xu, Muhammad Irfan

**Affiliations:** 1 National Navel Orange Engineering Research Center, School of life Sciences, Gannan Normal University, Ganzhou 341000, Jiangxi, China Gannan Normal University Ganzhou China; 2 School of Life Sciences, Guizhou Normal University, Guiyang 550025, China Guizhou Normal University Guiyang China; 3 Key Laboratory of Eco-environments in Three Gorges Reservoir Region (Ministry of Education), School of Life Sciences, Southwest University, Chongqing 400715, China Southwest University Chongqing China

**Keywords:** Copulatory organ, morphology, sheet-web spiders, Southeast Asia, taxonomy

## Abstract

*Floroniahuishuiensis* Zhou & Xu, **sp. nov.** (♂♀) is the first species in the genus *Floronia* to be described from Baiyan Cave in Guizhou Province, China. The new species is similar to *F.zhejiangensis* Zhu, Chen & Sha, 1987 but differs in structural details of the genital organs, primarily by the presence of a well-developed retrolateral tibial apophysis, a hook-shaped distal end of the radix in the male palp, and the rectangular posterior median plate in the epigyne. The illustration of copulatory organs of *F.bucculenta* (Clerck, 1757) and *F.zhejiangensis* Zhu, Chen & Sha, 1987 were reproduced here for comparison. A detailed description, photographs of the habitus and copulatory organs of the new species and a distribution map is provided.

## ﻿Introduction

Linyphiidae is the second largest family of relatively small spiders, with 4,832 species in 636 genera, which are commonly distributed across the globe, and including 517 species in 179 genera reported from China (World Spider Catalog 2023). The genus *Floronia* Simon, 1887 comprises six species distributed in China, Europe, Japan, Korea, and Russia (World Spider Catalog 2023).

With the addition of the new species described here, *Floroniahuishuiensis* Zhou & Xu, sp. nov., the number of *Floronia* species from China reaches five. The type species of the genus, *F.bucculenta* (Clerck, 1757), has a wide distribution in Europe, Russia and has now spread to five provinces in China (Hebei, Jilin, Liaoning, Qinghai, and Yunnan). While examining spider specimens from Guizhou Province, we identified a new species of Linyphiidae, which is described here. The illustrations of copulatory organs of *F.bucculenta* (Clerck, 1757) and *F.zhejiangensis* Zhu, Chen & Sha, 1987 are reproduced from the master’s thesis of Dr Xu Xin and are presented here for comparison.

## ﻿Materials and methods

Specimens were collected by handpicking and preserved in 95% ethanol. After dissection, the epigyne was cleared in trypsin enzyme solution before examination and photography. The left male pedipalps were used for description and illustration. Specimens were examined and measured with an Olympus BX41 stereomicroscope. Photographs were taken with a Kuy Nice CCD mounted on an Olympus BX41 stereomicroscope and focus stacked using Helicon Focus v. 3.10. Maps were created using ArcMap v. 10.2 and modified using Adobe Photoshop CS6 Extended. Leg measurements are shown as total length (coxa, trochanter, femur, patella, tibia, metatarsus, tarsus). All measurements are given in millimeters (mm). Terminology and taxonomic descriptions follow Saaristo and Tanasevitch (1996). The specimens of *Floroniahuishuiensis* Zhou & Xu, **sp. nov.** are deposited in the Taxidermy Museum of Gannan Normal University, Ganzhou City, China (GNNU), and specimens of *F.bucculenta* and *F.zhejiangensis* are deposited at the College of Life Sciences, Hubei University, Wuhan.

The following abbreviations are used in the text and figures:

Somatic characters: **AER** – anterior eye row; **ALE** – anterior lateral eye; **AME** – anterior median eye; **AME–ALE** – the distance between AME and ALE; **AME–AME** – the distance between AMEs; **d** – dorsal; **PLE** – posterior lateral eye; **l** – lateral; **PME** – posterior median eye; **PME–PLE** – distance between PME and PLE; **PME–PME** – distance between PMEs; **PER** – posterior eye row; **Tm** – trichobothrium; **v** – ventral.

Male pedipalp: **E** – embolus; **EP** – embolus proper; **LC** – lamella characteristica; **MM** – median membrane; **PC** – paracymbium; **PCA** – proximal cymbial apophysis; **PH** – pit hook on suprategulum; **R** – radix; **ST** – subtegulum; **T** – tegulum; **TA** – terminal apophysis; **TH** – thumb hook.

Epigyne: **PMP** – posterior median plate; **PS** – proscape; **S** – spermtheca; **St** – stretcher.

## ﻿Taxonomy

**Family Linyphiidae Blackwall, 1859**

**Subfamily Micronetinae Hull, 1920**

**Genus *Floronia* Simon, 1887**

### ﻿Key to the males of *Floronia* species from China

**Table:** 

1	Body size ≥2.5 mm and male pedipalp patella with one long spine	**2**
–	Body size ≤2.5 mm and male pedipalp patella with two spines	**4**
2	Pedipalp tibia with a long spine and three teeth on retrolateral margin	***F.bucculenta***
–	Pedipalp tibia with a long spine and one or no tooth on retrolateral margin	**3**
3	Outside of tibia with a dentation apophysis	***F.jiuhuensis***
–	Outside of tibia without a dentation apophysis	***F.hunanensis***
4	Tibia of pedipalp organ with a long spine and no distinct lateral apophysis	***F.zhejiangensis***
–	Tibia of pedipalp organ with a long spine, a dentation apophysis, and a distinct retrolateral tibia apophysis	***F.huishuiensis* Zhou & Xu, sp. nov.**

### ﻿Key to females of *Floronia* species from China

**Table:** 

1	Epigyne with a large lateral extension	**2**
–	Epigyne with a small lateral extension	***F.bucculenta***
2	Epigyne scapus wider on both sides	**3**
–	Epigyne scapus narrower on both sides and overlapping	***F.hunanensis***
3	Epigyne scapus folded and with an extension	***F.zhejiangensis***
–	Epigyne scapus folded and with tongue-shaped tip	***F.huishuiensis* Zhou & Xu, sp. nov.**

#### ﻿ Floronia bucculenta

Taxon classificationAnimaliaAraneaeLinyphiidae

(Clerck, 1757)

7B12C7BF-7D72-516A-BAB4-8A98DA795000

Figs 1 , 7 (三角弗蛛)

Araneus bucculentus Clerck, 1757: 63, pl. 4, f. 1.
Frontina bucculenta —Simon 1884: 207; Dupérré 2013: 7, fig. 55 (♂). For a full list of publications and synonyms of this species, see World Spider Catalog (2023).

##### Materials examined.

2♀1♂, China, Jilin Province, Changbai Mountain National Nature Reserve, 13 Aug. 1985.

**Figure 1.**
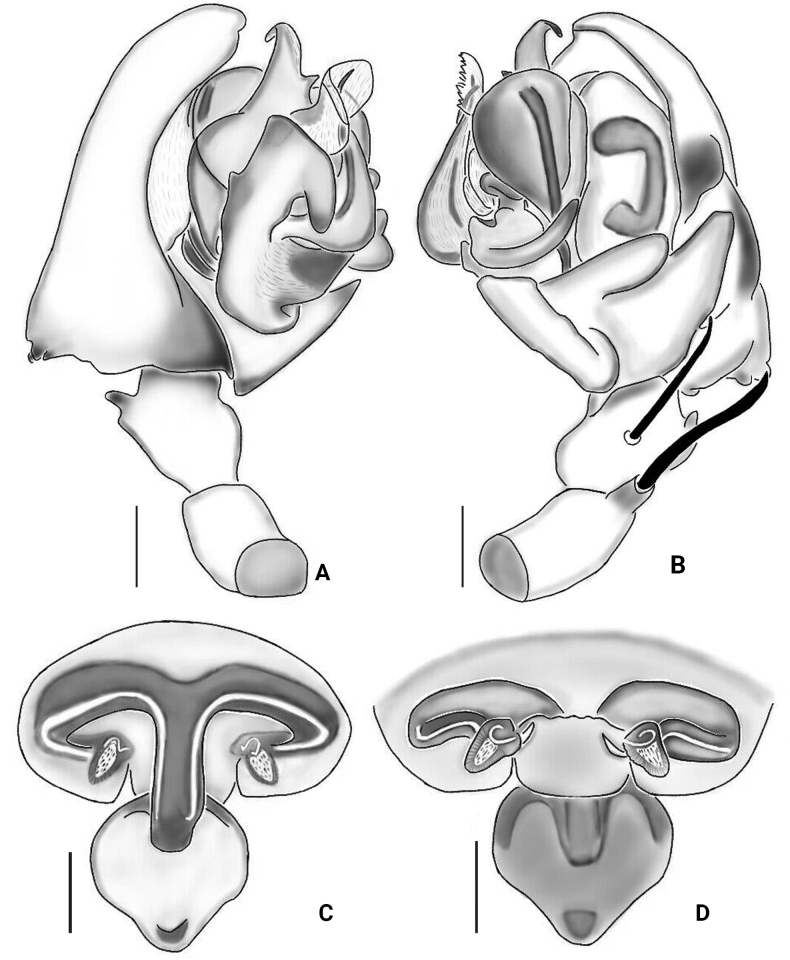
*Floroniabucculenta* (Clerck, 1757) **A, B** male pedipalp **C, D** female epigyne **A** prolateral view **B** retrolateral view **C** ventral view **D** dorsal view. Scale bars: 0.2 mm.

##### Distribution.

Europe to the Russian Far East; China (Fig. 7).

#### ﻿ Floronia huishuiensis

Taxon classificationAnimaliaAraneaeLinyphiidae

Zhou & Xu sp. nov.

66372D0B-D3C5-5F8C-9491-9BE9969A3CD8

https://zoobank.org/20721601-D222-4DED-8F44-257326320E92

**Figure 2.**
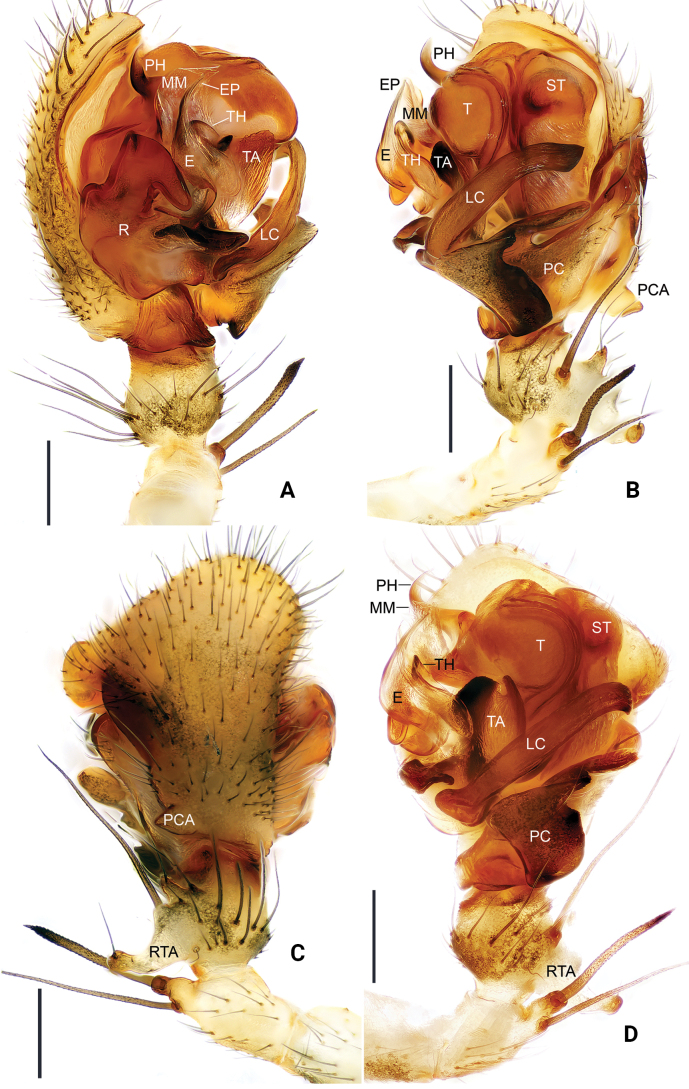
*Floroniahuishuiensis* Zhou & Xu, sp. nov., male holotype pedipalp **A** prolateral view **B** retrolateral view **C** dorsal view **D** ventral view. Scale bars: 0.2 mm.

**Figure 3.**
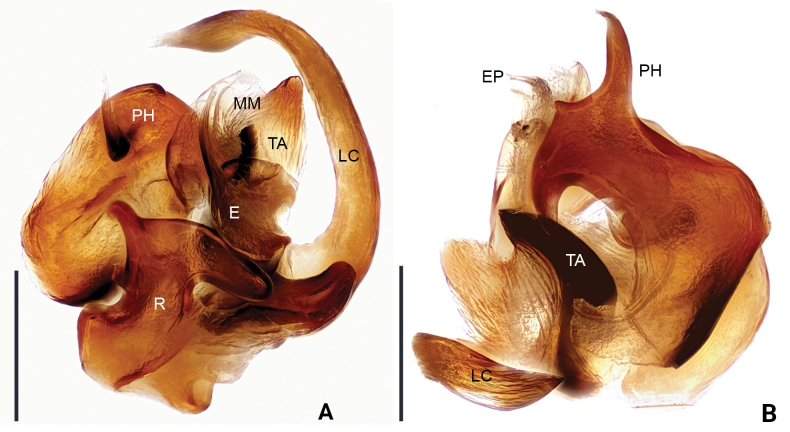
*Floroniahuishuiensis* Zhou & Xu, sp. nov., male pedipalp (one paratype, embolic division) **A** prolateral view **B** retrolateral view. Scale bars: 0.2 mm.

**Figure 4.**
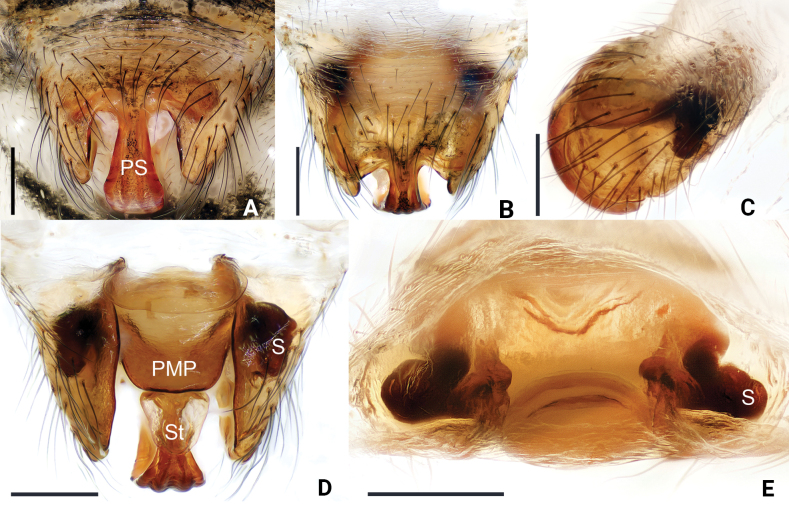
*Floroniahuishuiensis* Zhou & Xu, sp. nov., female paratype epigyne **A, B** ventral view **C** lateral view **D** dorsal view **E** anterior view. Scale bars: 0.2 mm.

**Figure 5.**
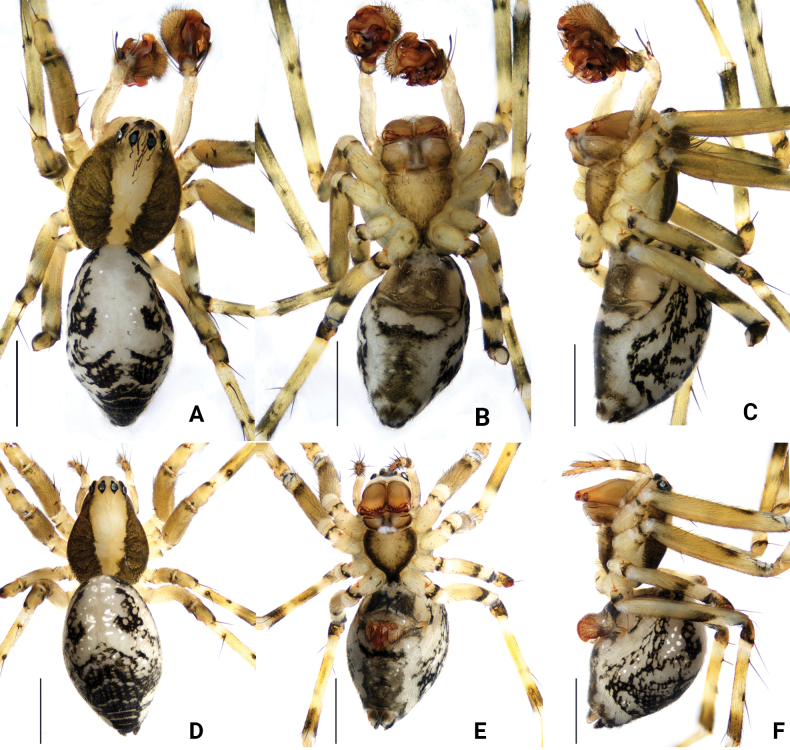
*Floroniahuishuiensis* Zhou & Xu, sp. nov., habitus **A–C** male holotype **D–F** female paratype **A, D** dorsal view **B, E** ventral view **C, F** lateral view. Scale bars: 1 mm.

Figs 2 , 3 , 4 , 5 , 7 (惠水弗蛛)

##### Type materials.

***Holotype*** ♂, China: Guizhou Province: Huishui County, Baiyan Cave has light bands. 25.9582°N, 106.6423°E, alt. 1158 m, 07 Oct. 2020, Weifeng Du, Siqiang Zhang, Rui Zuo and Xuemei Jiang leg. (GNU-BYC-20-01-05-1). ***Paratypes***: 1 ♂2♀, collected with the holotype (GNU-BYC-20-01-05-2 to GNU-BYC-20-01-05-4).

##### Etymology.

The specific epithet is derived from the name of the county where the type locality is located. Gender neutral.

##### Diagnosis.

**Figure 6.**
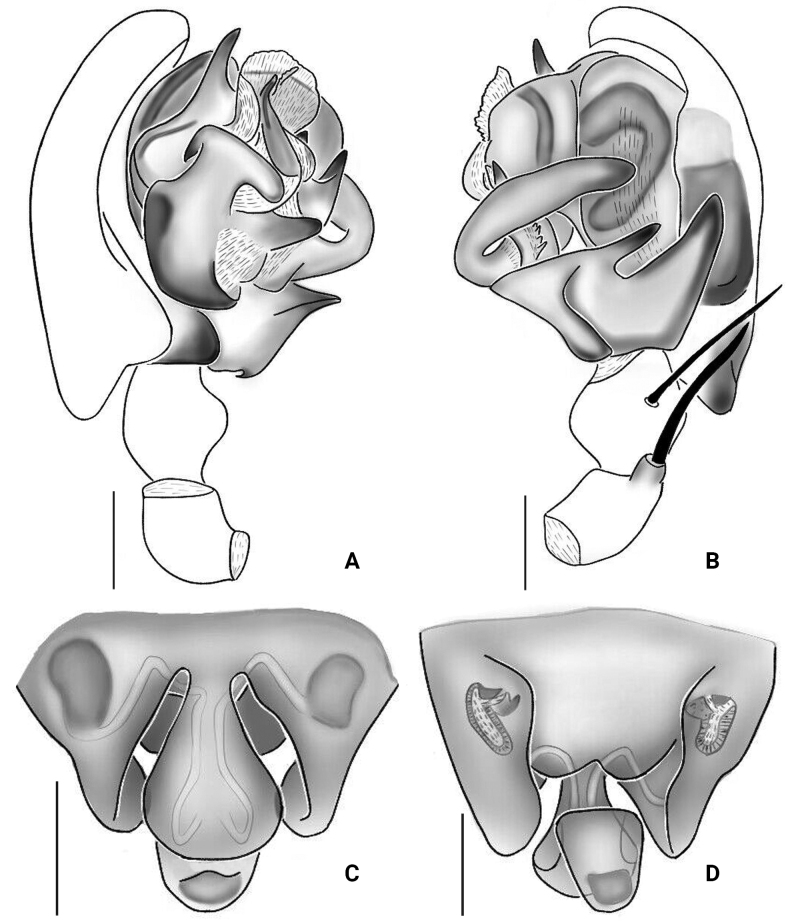
*Floroniazhejiangensis* Zhu, Chen & Sha, 1987 **A, B** male pedipalp **C, D** female epigyne **A** prolateral view **B** retrolateral view **C** ventral view **D** dorsal view. Scale bars: 0.2 mm.

This new species resembles *F.zhejiangensis* Zhu, Chen & Sha, 1987 in having the similar morphology of cephalothorax, pedipalp with long lamella characteristca, and epigyne with similar proscape (Figs 2–4, 6), but it can be distinguished by the following: (1) tibia with well-developed retrolateral apophysis in male pedipalp Fig. 2A, B) vs absent in *F.zhejiangensis* (Fig. 6A, B); (2) distal end of radix hook-shaped in prolateral view (Fig. 2A) vs anchor-shaped in *F.zhejiangensis* (Fig. 6A, B). Females can be distinguished by having the posterior median plate rectangular s (Fig. 4D) vs somewhat trapezoid in *F.zhejiangensis* (Fig. 6D).

##### Description.

**Male** (holotype, Fig. 5A−C). Total length 1.51. Carapace 0.65 long, 0.52 wide, with a broad, longitudinal, white band at centre and sides greenish-brown sides. Abdomen 0.86 long, 0.51 wide. Anterior half of abdomen mostly white; posterior half with dark pattern. Sternum 0.53 long, 0.52 wide. Clypeus 0.11 high. Chelicerae with seven promarginal and six retromarginal teeth. Eye sizes and interdistances: AME 0.07, ALE 0.08, PME 0.07, PLE 0.07, AME–AME 0.03, AME–ALE 0.05, PME–PME 0.07, PME–PLE 0.06, AME–PME 0.12, ALE–PLE 0.01. Spination: tibiae I–IV d2222, l2010, v2112; metatarsi I–IV d1222 lateral and ventral spines absent. Length of legs: I 4.20 (0.46, 0.11, 0.87, 0.19, 0.89, 1.14, 0.54), II 3.61 (0.41, 0.09, 0.77, 0.17, 0.73, 0.99, 0.45), III 2.40 (0.35, 0.17, 0.45, 0.15, 0.41, 0.55, 0.32), IV 3.23 (0.41, 0.18, 0.65, 0.15, 0.61, 0.82, 0.41). TmI 0.35, TmIV 0.25.

Pedipalp (holotype, Fig. 2A−D, one paratype, Fig. 3A, B). Patella as long as tibia, retrolaterally with two large macrosetae; tibia with one retrolateral and one dorsal trichobothria; retrolateral tibial apophysis well developed, doorknob-like in dorsal view, with blunt end. Cymbium retrolateral margin with a shallow depression at base; prolateral margin with a small tubercle; proximal cymbial apophysis somewhat thumb-shaped in dorsal view. Paracymbium with well-developed anterior and distal arms; prolateral margin longer than wide, apical part rod-like with blunt end. Distal suprategulum with large, slightly curved pit-hook; median membrane well developed, with serrated margin. Radix tip hook-shaped; lamella characteristca simple, semicircular; terminal apophysis apically widened, with bifurcated tip. Embolus flame-lake. Embolus proper set apically, with serrated margin; thumb slightly curved, almost as long as embolus.

**Female** (paratype, Fig. 5D−F). Total length 1.59. Carapace 0.67 long, 0.47 wide. Abdomen 0.92 long, 0.63 wide. Sternum 0.28 long, 0.34 wide. Clypeus 0.01 high. Chelicerae with seven promarginal and six retromarginal teeth. Eye sizes and interdistances: AME 0.07, ALE 0.07, PME 0.06, PLE 0.07, AME–AME 0.03, AME–ALE 0.03, PME–PME 0.06, PME–PLE 0.06, AME–PME 0.11, ALE–PLE 0.01. Spination: tibiae I–IV d2222, l2201, v2201; metatarsi I–IV d2222 lateral and ventral spines absent. Length of legs: I 4.09 (0.48, 0.22, 0.73, 0.19, 0.88, 1.03, 0.56), II 3.53 (0.42, 0.20, 0.65, 0.15, 0.74, 0.89, 0.48), III 2.35 (0.36, 0.14, 0.53, 0.11, 0.41, 0.55, 0.25), IV 3.06 (0.41, 0.20, 0.65, 0.15, 0.63, 0.71, 0.31). TmI and TmIV present. Cephalothorax and abdominal colour pattern same as in male.

##### Epigyne.

(Fig. 4A−E) Proscape longer than wide; stretcher oval, translucent; lateral lobes of proscape inconspicuous. Posterior median plate somewhat rectangular. Spermathecae present anterolaterally.

##### Distribution.

**Figure 7.**
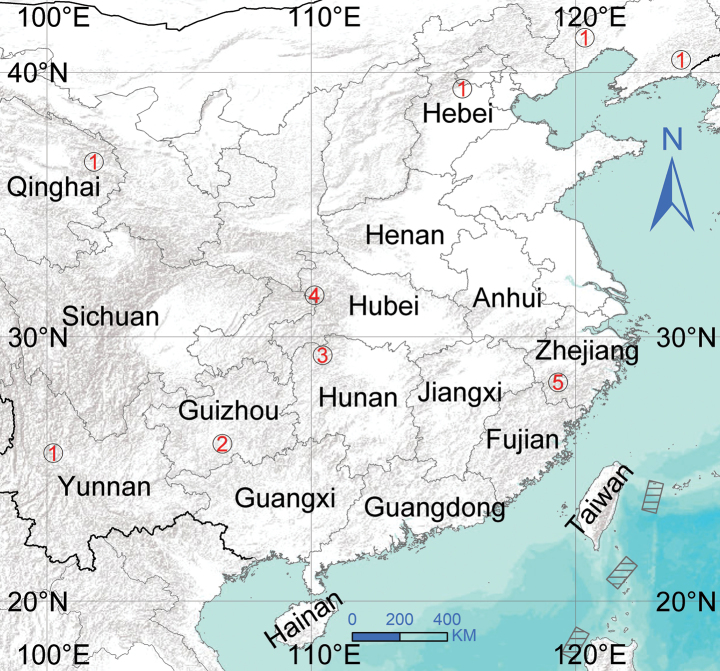
Distribution of *Floronia* species in China. 1 = *Floroniabucculenta* (Clerck, 1757), 2 = *F.huishuiensis* Zhou & Xu, sp. nov., 3 = *F.hunanensis* Li & Song, 1993, 4 = *F.jiuhuensis* Li & Zhu, 1987, 5 = *F.zhejiangensis* Zhu, Chen & Sha, 1987.

Known only from the type locality in Guizhou, China (Fig. 7).

##### Habitat.

Baiyan Cave is a natural karst cave with a large opening. The new species, which was mainly found about 10–20 m from the cave’s opening, makes webs under large rocks.

#### ﻿ Floronia zhejiangensis

Taxon classificationAnimaliaAraneaeLinyphiidae

Zhu, Chen & Sha, 1987

13198FCA-E315-5574-A77B-62F0A86E0BDE

Figs 6 , 7 (浙江弗蛛)

Floronia zhejiangensis Zhu, Chen & Sha, 1987: 139, figs 1−8. For full list of publications and synonyms concerning this species, see World Spider Catalog (2023).

##### Materials examined.

2♂3♀, China, Zhejiang Province, Anmin Township, Songyang County, Lishui City, 28.28154°N, 119.33576°E, 520 m, 10 Jan. 1986, Yinfang Chen leg.

##### Distribution.

China (Fig. 7).

## Supplementary Material

XML Treatment for Floronia bucculenta

XML Treatment for Floronia huishuiensis

XML Treatment for Floronia zhejiangensis

## ﻿Additional information

### Author ORCIDs

Guchun Zhou https://orcid.org/0000-0002-5757-0317

Weifeng Du https://orcid.org/0009-0007-4971-4206

Chengxiang Xu https://orcid.org/0000-0002-7228-3981

Muhammad Irfan https://orcid.org/0000-0003-0445-9612

### Data availability

All of the data that support the findings of this study are available in the main text.
